# Involvement of Two Paralogous Methoprene-Tolerant Genes in the Regulation of Vitellogenin and Vitellogenin Receptor Expression in the Rice Stem Borer, *Chilo suppressalis*

**DOI:** 10.3389/fgene.2020.00609

**Published:** 2020-06-10

**Authors:** Lijun Miao, Nan Zhang, Heng Jiang, Fan Dong, Xuemei Yang, Xin Xu, Kun Qian, Xiangkun Meng, Jianjun Wang

**Affiliations:** ^1^College of Horticulture and Plant Protection, Yangzhou University, Yangzhou, China; ^2^Joint International Research Laboratory of Agriculture and Agri-Product Safety of the Ministry of Education, Yangzhou University, Yangzhou, China

**Keywords:** *Chilo suppressalis*, juvenile hormone, methoprene-tolerant, vitellogenin, vitellogenin receptor, RNAi

## Abstract

Besides the function of preventing metamorphosis in insects, the juvenile hormone (JH) plays a role in female reproduction; however, the underlying mechanism is largely unknown. The methoprene-tolerant (Met) protein belongs to a family of basic helix-loop-helix–Per-Arnt-Sim (bHLH-PAS) transcription factors and functions as the JH intracellular receptor. In this study, two full length cDNAs encoding *Met* (*CsMet1* and *CsMet2*) were isolated from the rice stem borer, *Chilo suppressalis.* Structural analysis revealed that both *CsMet1* and *CsMet2* exhibited typical bHLH, PAS-A, PAS-B, and PAC (PAS C terminal motif) domains. Comparative analysis of transcript level using reverse transcription-quantitative PCR (RT-qPCR) revealed that *CsMet1* was predominant in almost all examined developmental stages and tissues. Treatment with methoprene *in vivo* induces the transcription of both *CsMet1* and *CsMet2.* Notably, injection of dsCsMet1 and dsCsMet2 suppressed the expression levels of vitellogenin (*CsVg*) and Vg receptor (*CsVgR*). These findings revealed the potential JH signaling mechanism regulating *C. suppressalis* reproduction, and provided evidence that RNAi-mediated knockdown of *Met* holds great potential as a control strategy of *C. suppressalis.*

## Introduction

The rice stem borer, *Chilo suppressalis* (Walker) (Lepidoptera: Crambidae), is one of the most serious rice pests in Asia, Middle East, and southern Europe, and causes large crop losses through feeding on the stems of rice. In China, the change of cultivation patterns has led to the frequent outbreaks of *C. suppressalis* in recent years (Mao et al., 2019). To date, spraying chemical insecticides remains the primary strategy for controlling *C. suppressalis*. However, intensive use of insecticides has driven *C. suppressalis* to develop resistance to a wide range of insecticides (Su et al., 2014; Lu et al., 2017; Yao et al., 2017). Hence, the development of RNA interference (RNAi)-mediated disruption of reproduction represents an alternative control strategy of *C. suppressalis* (Miao et al., 2020).

Insect reproduction is intricately regulated by three hormones, including the juvenile hormones (JHs), ecdysteroids, and insulin (Smykal and Raikhel, 2015; Roy et al., 2018; Al Baki et al., 2019). JH exerts its function through its intracellular receptor methoprene-tolerant (Met), a member of the family of the basic helix-loop-helix (bHLH)-Per-Arnt-Sim (PAS) transcription factors (Konopova and Jindra, 2007; Jindra et al., 2013, 2015). Since the first characterization of Met in *Drosophila melanogaster* (Wilson and Fabian, 1986), Met has been identified from a broad range of insect species, such as *Aedes aegypti* (Wang et al., 2007), *Tribolium castaneum* (Konopova and Jindra, 2007), *Nilaparvata lugens* (Lin et al., 2015), and *Helicoverpa armigera* (Ma et al., 2018). Interestingly, two Met paralogs, Met and Germ cell-expressed (Gce), are present across 12 *Drosophila* species (Baumann et al., 2010a, b). While mosquitoes and beetles possess only a single gene, two *Met* genes have also been identified in several Lepidopteran insects, including *Danaus plexippus* (Zhan et al., 2011), *Operophtera brumata* (Derks et al., 2015), and *Bombyx mori* (Guo et al., 2012; Suetsugu et al., 2013; Kayukawa and Shinoda, 2015). However, the role of two *Met* genes in the reproduction of Lepidopteran insects remains largely unknown.

The reproductive success of insects depends on vitellogenin (Vg) biosynthesis and uptake of Vg into developing oocytes mediated by vitellogenin receptor (VgR). Recently, we have characterized the *C. suppressalis CsVg* and *CsVgR* at the molecular levels (Huang et al., 2016; Miao et al., 2020). In this study, full-length cDNAs of two paralogous *Met* genes (named as *CsMet1* and *CsMet2*) were isolated from *C. suppressalis*. We report the structural features and temporal–spatial expression patterns of *CsMet1* and *CsMet2.* Furthermore, RNAi was employed to reveal the role of *CsMet1* and *CsMet2* in the regulation of *CsVg* and *CsVgR*.

## Materials and Methods

### Insects Rearing and Sampling

*Chilo suppressalis* larvae was collected from rice stubbles of Yangzhou (32.39°N, 119.42°E) in 2013, and reared on an artificial diet in an incubator at 28 ± 1°C, 70 ± 5% RH, and a 16-h light/8-h dark photoperiod.

To examine the developmental expression profiles of *CsMet*, individuals were collected from larvae (third, fourth, fifth, and sixth instar), pupae at intervals of 2 days from pupation, and adults at intervals of 12 h from eclosion. For tissue expression analysis, tissues (including head, epidermis, midgut, hemolymph, and fat body) were dissected from the fifth-instar larva. All the samples were frozen immediately in liquid nitrogen and stored at -80°C until RNA isolation. Each experiment was performed with three biological replicates containing three to 10 individuals.

### RNA Isolation, RT-PCR, and RACE

Total RNA was extracted using Trizol reagent (Invitrogen, Carlsbad, CA, United States). First-strand cDNA was synthesized from total RNA using the Primescript^TM^ First-Strand cDNA Synthesis Kit (TaKaRa, Dalian, China) according to the manufacturers’ instruction.

The amino acid (aa) sequences of *B. mori BmMet1* (GenBank: NP001108458) and *BmMet2* (GenBank: BAJ05086) were searched against the transcriptome database of *C. suppressalis* in Insect Base^1^, and specific primer pairs were designed based on the sequences of putative transcripts (Table 1). PCR reactions were performed with LA Taq^TM^ DNA polymerase (TaKaRa, Dalian, China). To obtain full length cDNA sequences of *CsMet1* and *CsMet2*, 5′-RACE and 3′-RACE were conducted using the SMART RACE cDNA Amplification Kit (Clontech, Mountain View, CA, United States), following the manufacturers’ instructions. Gene specific primers (GSPs) used for RACE are listed in Table 1.

**TABLE 1:** Oligonucleotide primers used for RT-PCR, RACE, RT-qPCR, and RNAi.

Primer name	Sequence (5′ to 3′)	Description
W988Met1 F	ATGACATCATTGACTGGAGC	*CsMet1* RT-PCR
W989Met1 R	GGATTACAGGATTTCAGTTTCTGA	
W71Met1 R	CAACAGGCAGTCAGTCACCAAGT	*CsMet1* 5′-RACE
W72Met1 R	TGACATCATTGACTGGAGCCACTG	
W73Met1 F	TGTTGGTGTAGATTATGGGCGACG	*CsMet1* 3′-RACE
W74Met1 F	AAGTGGTGCAAGAAACTGGTGTCC	
W990Met2 F	AGAGAGATTCGAAACAAAGCG	*CsMet2* RT-PCR
W991Met2 R	TCGTTTGTACCAACACTGTC	
W75Met2 R	CCAGCATTTCGGCGACCTTGTTCTT	*CsMet2* 5′-RACE
W76Met2 R	CCAGTTCACCGATGGATTGGTTCAG	
W77Met2 F	GTGTTCGTGGGCATTGTCCGCTTGG	*CsMet2* 3′-RACE
W78Met2 F	TTTAGTCGGTGAGTCCTGCTATCGT	
EF1-α F	TGAACCCCCATACAGCGAATCC	RT-qPCR
EF1-α R	TCTCCGTGCCAACCAGAAATAGG	
W465Met1 qF	TGGCTTCCTCGAGATTGACA	RT-qPCR
W466Met1 qR	TCCTGATGCTACCCCAGATG	
W469Met2 qF	CAATCCATCGGTGAACTGGC	RT-qPCR
W470Met2 qR	GTTGAGTATGGACAGCAGCG	
W84VgR F	AGCCACTTCCCTACCTCCTA	RT-qPCR
W85VgR R	TAAGGCATTGGGGACTCGTT	
W976Vg F	AGCTCAGTCCGCTAAATGGA	RT-qPCR
W977Vg R	GCCCAGTTCGTGGTGTCTAT	
W463Met1 iF	TAATACGACTCACTATAGGGTCTGAC ATAGTGCACGCTCC	*CsMet1* RNAi
W464Met1 iR	TAATACGACTCACTATAGGGTGTGCG TACCCTTCTGACAC	
W467Met2 iF	TAATACGACTCACTATAGGGCAGGGG GCTCATTGTGGTAG	*CsMet2* RNAi
W468Met2 iR	TAATACGACTCACTATAGGGGTGCCG CGTTCTATACTCCA	

### Molecular Cloning and Sequence Analysis

RT-PCR and RACE products were subcloned into the pMD18−T vector (TaKaRa) and sequenced. The molecular mass and isoelectric point (pI) of the deduced protein sequences were predicted by using the online ExPASy proteomics server^2^. Conserved domains were predicted by NCBI conserved domain search tool^3^ or by alignment to other published insect Mets. A phylogenetic tree was constructed with MEGA 7.0 using the neighbor-joining method with a *p*-distance model and a pairwise deletion of gaps (Kumar et al., 2016). The reliability of the NJ tree topology was statistically evaluated by bootstrap analysis with 1000 replicates.

### Reverse Transcription-Quantitative PCR (RT-qPCR)

Reverse transcription-quantitative qPCR reactions were performed on the Bio-Rad CFX-96TM Real-time PCR system using TB Green^TM^ Premix Ex Taq^TM^ (Takara, Dalian, China) following the manufacturers’ instructions. GSPs used are listed in Table 1. The stably expressed gene encoding EF1-α was used as a reference gene (Hui et al., 2011; Meng et al., 2019). The mRNA levels were normalized to reference gene with the 2^–ΔΔ*CT*^ method (Livak and Schmittgen, 2001). The means and standard errors for each time point were obtained from the average of three biologically independent samples.

### Methoprene Treatment

To study effects of JH on expression of *CsMet*, newly emerged adult females were injected with 1 μL JH analog methoprene (3 μg/μL, Sigma–Aldrich, St. Louis, MO, United States) or same volume of acetone as control. Insects were collected for RT-qPCR analysis at 1, 6, 12, and 24 h after treatment, respectively, and experiments contained three biological replications.

### RNA Interference

Double-strand RNAs (dsRNAs) against *CsMet1* or *CsMet2* were synthesized using TranscriptAid^TM^ T7 High Yield Transcription Kit (Thermo Fisher Scientific, Waltham, MA, United States). GSPs for dsRNA synthesis are listed in Table 1. Using a Nanoliter 2010 injector system (WPI, Sarasota, FL, United States), 1 μL solution of dsRNA (3 μg/μL) was injected into the abdomen of 6-day-old female pupae under a stereomicroscope. Equal dose of dsRNA for enhanced green fluorescent protein (EGFP) was injected as a control. A total of 40 pupae were injected per treatment, and each treatment was performed in triplicate. The treatment was repeated in newly emerged female adults (within 24 h), and insects were collected at 24 and 48 h after injection, respectively, for expression analysis of *CsMet1*, *CsMet2*, *CsVg*, and *CsVgR* by RT-qPCR.

### Data Analysis

The statistical analysis was done using graphpad.prism.6 by one-way analysis of variance, followed by student’s *t*-test. All data are presented as the mean ± SE.

## Results

### Sequence and Structural Analysis of *CsMet1* and *CsMet2*

The full length *CsMet1* cDNA (GenBank accession number MN906993) was 2673 bp, with a 460 bp 5’-terminal untranslated region (UTR), a 1587 bp open reading frame (ORF), and a 626 bp 3′-UTR. The deduced *CsMet1* protein contained 528 aas with a predicted mass and pI of 60.34 kDa and 8.35, respectively. The 2824 bp *CsMet2* cDNA (GenBank accession number MN906994) contained an 85 bp 5′-UTR, a 2598 bp ORF encoding an 865 aa residue protein with a molecular mass of 97.37 kDa and a pI of 6.68, and a 141 bp 3′-UTR.

**FIGURE 1:**
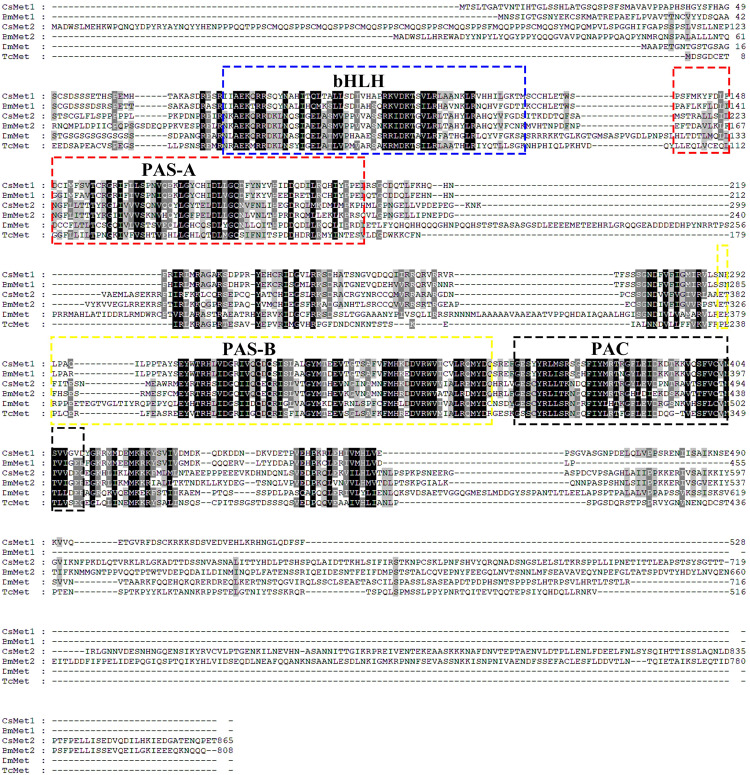
Amino acid sequence alignments of *CsMet1*, *CsMet2* and other representative insect Mets. Met sequences were obtained from the following GenBank entries: NP001108458 for *B. mori BmMet1*, BAJ05086 for *B. mori BmMet2*, AAC14350 for *D. melanogaster DmMet* and NP 001092812 for *T. castaneum TcMet*.

**FIGURE 2:**
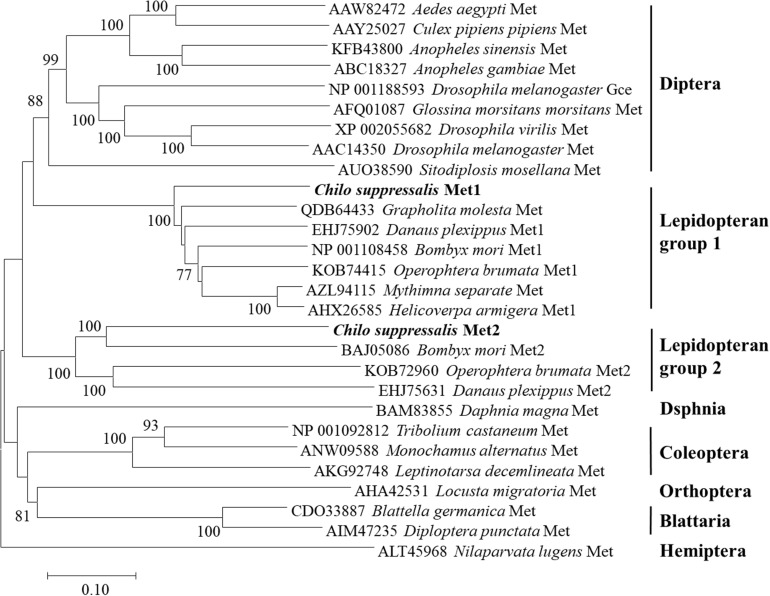
Phylogenetic relationships of *CsMet1*, *CsMet2* and other insect Mets. The neighbor-joining tree was constructed by MEGA 7.0. Numbers indicated bootstrap support values (%) based on 1,000 replicates. Sequences were retrieved from GenBank protein database.

Structural analysis showed that the deduced protein of *CsMet1* and *CsMet2* had conserved domains of bHLH-PAS protein family including bHLH (DNA binding and dimerization regions), PAS-A (dimerization region), PAS-B (ligand binding and dimerization region), and PAC (PAS C terminal motif) domains (dimerization region) (Figure 1). Alignment of aa sequences demonstrated that *CsMet1* shared identity with other insect Met orthologs including *B. mori BmMet1* (60.98%) and *BmMet2* (21.88%), *D. melanogaster DmMet* (27.49%), and *T. castaneum TcMet* (28.30%). *CsMet2* shared 20.55, 44.19, 17.19, and 22.90% identity with *BmMet1*, *BmMet2*, *DmMet*, and *TcMet*, respectively. The aa identity between *CsMet1* and *CsMet2* was 21.71% (Figure 1). Phylogenetic analysis revealed that *CsMet1* and *CsMet2* were clustered into Lepidopteran group 1 and group 2 Met, respectively (Figure 2).

### Temporal and Spatial Expression of *CsMet1* and *CsMet2*

**FIGURE 3:**
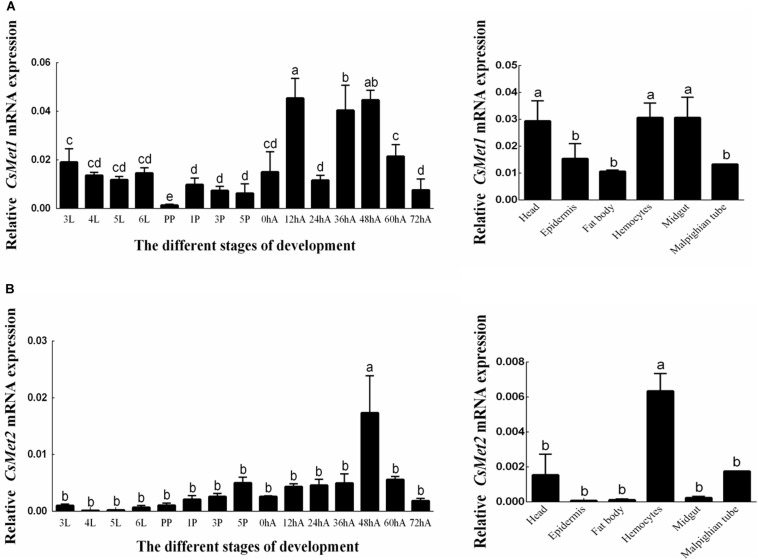
Expression profiles of *CsMet1* **(A)** and *CsMet2* **(B)** in different developmental stages and various tissues from 5^th^ instar larva. 3L: 3^3rd^ instar larvae; 4L: 4^th^ instar larvae; 5L: 5^th^ instar larvae; 6L: 6^th^ instar larvae; PP: prepupae; 1P: 1-day-old pupae; 3P: 3-day-old pupae; 5P: 5-day-old pupae; 0hA: newly emerged female adults; 12hA: 12-h-old female adults; 24hA: 24-h-old female adults; 36hA: 36-h-old female adults; 48hA: 48-h-old female adults; 60hA: 60-h-old female adults; 72hA: 72-h-old female adults. Bars not sharing a common letter are significantly different.

The temporal expression patterns of *CsMet1* and *CsMet2* were detected from third instar to 3-day-old female adults and determined by RT-qPCR analysis. The results showed that the transcription level of *CsMet1* and *CsMet2* was detectable during all selected developmental stages. Specifically, the expression of *CsMet1* was relatively stable in the larval stage, sharply decreased in the prepupal stage, maintained at a low level during the pupal stage, and reached a peak in 12-h-old female adults (Figure 3A). The expression of *CsMet2* gradually increased from fourth instar to 5-day-old pupae. In the adult stage, the highest and lowest expression levels of *CsMet2* were observed in 48-h-old and 72-h-old female adults, respectively (Figure 3B).

Analysis of the spatial expression profiles of *CsMet1* and *CsMet2* in fifth-instar larva showed that *CsMet1* was highly expressed in head, hemocytes, and midgut, while relatively low expression was observed in epidermis, fat body, and malpighian tube (Figure 3A). The highest expression level of *CsMet2* was observed in hemocytes, followed by head and Malpighian tube, whereas low expression was observed in epidermis, fat body, and midgut (Figure 3B).

### Effect of JHA Treatment

**FIGURE 4:**
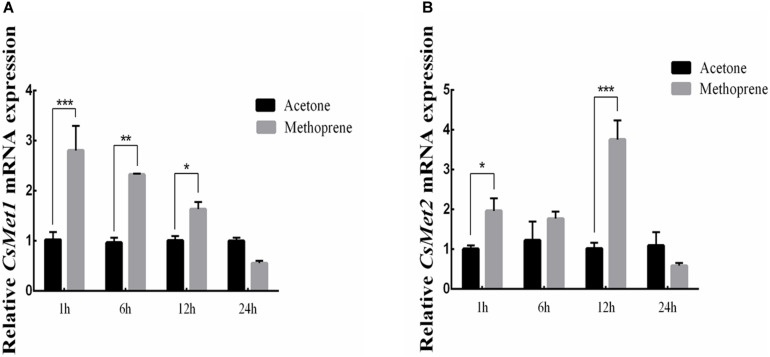
Relative mRNA levels of *CsMet1* **(A)** and *CsMet2* **(B)** in the whole body insects after methoprene treatment in newly emerged female adults. The bars represent mean ± SE of three biological replicates. Asterisks indicate the significant differences between the groups (*P < 0.05, **P < 0.01, and ***P < 0.001).

To examine whether *CsMet1* and *CsMet2* were regulated by JH, JHA methoprene was injected into the newly emerged female adults. Compared with control, the results showed that *CsMet1* expression was upregulated by 2.74, 2.41, and 1.63 times at 1, 6, and 12 h after injection, respectively (Figure 4A). Similarly, transcription level of *CsMet2* was increased by 1.95, 1.45, and 3.68 times at 1, 6, and 1 2 h post-treatment, respectively (Figure 4B).

### Effects of RNAi-Mediated Knockdown of *CsMet1* and *CsMet2*

**FIGURE 5:**
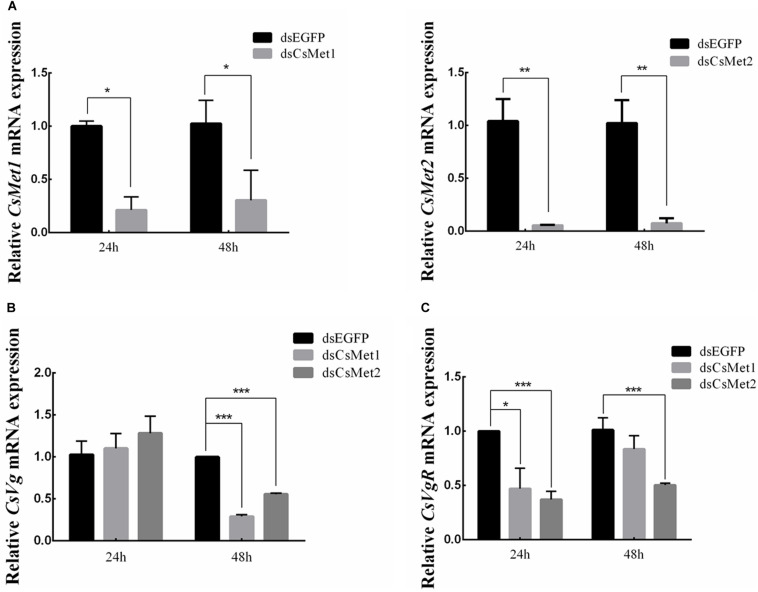
Knockdown of *CsMet1* and *CsMet2*. dsRNAs were injected into 6-day-old female pupae followed by the second injection into the newly emerged female moths. The expression levels of *CsMet1*, *CsMet2* **(A)**, *CsVg* **(B)**, and *CsVgR* **(C)** in whole body insects were examined at 24 h and 48 h after treatment. The bars represent mean ± SE of three biological replicates. Asterisks indicate the significant differences between the groups (*P < 0.05, **P < 0.01, and ***P < 0.001).

**FIGURE 6:**
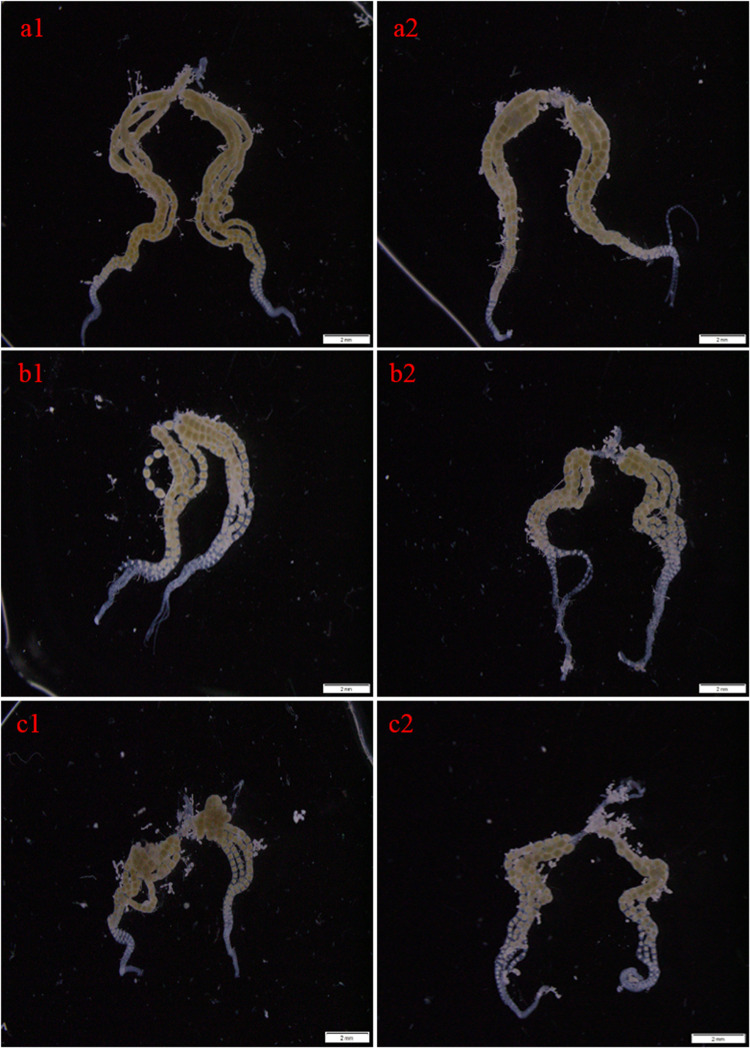
Effects of RNAi-mediated knockdown of *CsMet1* and *CsMet2* on ovary development with dsEGFP as a control. The ovaries were dissected from female adults at 24 h after the second injection of dsEGFP (a1, a2), dsCsMet1 (b1, b2) and dsCsMet2 (c1, c2).

To explore the role of *CsMet1* and *CsMet2* in the regulation of *CsVg* and *CsVgR*, female adults were collected at 24 and 48 h after the second dsRNA injection, respectively. The transcript level of *CsMet1* in dsCsMet1-injected female adults was suppressed by 78.74% (24 h) and 70.32% (48 h) compared with dsEGFP-injected insects, whereas the expression of *CsMet2* was significantly reduced by 94.69% (24 h) and 92.79% (48 h) (Figure 5A). Meanwhile, RNAi-mediated suppression of *CsMet1* and *CsMet2* decreased *CsVg* expression by 70.85 and 44.37% at 48 h post-treatment, respectively (Figure 5B). *CsVgR* expression in the dsCsMet1-treated and dsCsMet2-treated group decreased significantly by 52.91 and 62.98% at 24 h post-injection, respectively (Figure 5C). Furthermore, phenotypic observation revealed that silencing of both *CsMet1* and *CsMet2* resulted in suppressed ovarian development with decreased number of mature follicles (Figure 6).

## Discussion

The important roles played by JH in insect development and reproduction prompted the study on JH signaling, and the breakthrough had been the identification of transcription factor Met as an intracellular receptor for JH (Charles et al., 2011). Binding of JH to Met triggers dimerization of Met with another bHLH-PAS protein Taiman (Tai) to form a functional complex, which interacts with JH response elements (JHREs) of target genes (Smykal and Raikhel, 2015). While the *Met* null allele was expected to result in a lethal phenotype, the paralogous *Met* gene in *D. melanogaster*, *Gce*, ensured survival of the met null mutants (Baumann et al., 2010a, b). Similarly, two *Met* genes were also recently identified in several Lepidopteran insects (Zhan et al., 2011; Guo et al., 2012; Suetsugu et al., 2013; Derks et al., 2015; Kayukawa and Shinoda, 2015). However, only a single *Met* gene was found in those from other insect orders including *A. aegypti* (Dipteran, Wang et al., 2007), *T. castaneum* (Coleopteran, Konopova and Jindra, 2007), *Blattella germanica* (Blattarian, Lozano and Belles, 2014), *N. lugens* (Hemipteran, Lin et al., 2015), *Sitodiplosis mosellana* (Dipteran, Cheng et al., 2018), and so on. In this study, we isolated two paralogous *Met* genes, *CsMet1* and *CsMet2*, from *C. suppressalis*. Both proteins showed conserved domain organization of bHLH-PAS protein family (Ashok et al., 1998; Kayukawa and Shinoda, 2015; Ma et al., 2018). Phylogenetic analysis revealed that *CsMet1* and *CsMet2* were clustered into Lepidopteran group 1 and group 2 Met, respectively, providing further evidence of the occurrence of *Met* gene duplication in Lepidopteran insects.

The temporal and spatial expression patterns of a gene may provide hints as to its function. In this study, we found a marked reduction in expression levels of *CsMet1* in the prepupae stage, supporting its involvement in larval–pupal metamorphosis. Similar results were also observed in *HaMet1* of *H. armigera* (Ma et al., 2018) and *SmMet* of *S. mosellana* (Cheng et al., 2018), and was in agreement with the fact that JH exerts its anti-metamorphic effect through its receptor Met (Parthasarathy et al., 2008; Li et al., 2019). *CsMet2* transcript was hardly detected during the larval stage, and its expression reached the peak in 48-h-old female adults, suggesting that *CsMet2* was involved in JH signaling in adults, as had been reported for *BmMet2* in *B. mori* (Kayukawa and Shinoda, 2015). For tissue expression, *CsMet1* was highly expressed in larval head, midgut, and hemocytes, whereas *CsMet2* showed the highest expression in larval hemocytes. Interestingly, the temporal and spatial expression analysis revealed that *CsMet1* was dominant at almost all developmental stages and in all tissues examined. This result was somewhat different from the observation in *B. mori* that *BmMet2* transcript was more abundant than *BmMet1* transcript in the adult stage (Kayukawa and Shinoda, 2015). Whether *CsMet1* played a more important role in *C. suppressalis* needed further study in the future.

Juvenile hormone plays a crucial role in insect reproduction, but its molecular mode of action only became clear recently. It has been reported in *H. armigera*, *Diploptera punctata*, *N. lugens*, and S. *mosellana* that *Met* is significantly upregulated by JH III or JHA methoprene (Marchal et al., 2014; Zhao et al., 2014; Lin et al., 2015; Cheng et al., 2018). Our hormone treatment results also indicated that *CsMet1* and *CsMet2* expression was upregulated after JHA methoprene treatment. Further RNAi analysis revealed that knockdown of *CsMet1* and *CsMet2* resulted in significant decline of both *CsVg* and *CsVgR* expression in female adults, leading to decreased number of mature follicles in ovaries. Similarly, expression of *Vg* is regulated by JH via *Met* in *A. aegypti*, *H. armigera*, *D. punctata*, *N. lugens*, and *Bactrocera dorsalis* (Zou et al., 2013; Marchal et al., 2014; Lin et al., 2015; Ma et al., 2018; Yue et al., 2018). Downregulation of *VgR* transcription as well as suppressed ovarian development were also observed in dsMet-treated *H. armigera* and *Colaphellus bowringi* (Ma et al., 2018; Liu et al., 2019). Whether expression of *Vg* and *VgR* were directly regulated by Met was not well known. It had been recently reported that knockdown of Krüppel-homolog 1 (Kr-h1), a direct target of JH-Met signaling, also decreased *VgR* expression in *C. bowringi* (Liu et al., 2019). On the other hand, Met is also a repressor of 20E pathway gene expression. It is likely that Met regulated the relative genes of 20E pathway (Zhao et al., 2014), which contributes to vitellogenis to some extent. Further study needs to be carried out to confirm these presumptions.

## Data Availability Statement

All datasets generated for this study are included in the article.

## Author Contributions

JW conceived and designed the study. LM, NZ, HJ, FD, XY, and XX performed the experiments. LM, JW, KQ, and XM wrote the manuscript. All authors reviewed and approved this submission.

## Conflict of Interest

The authors declare that the research was conducted in the absence of any commercial or financial relationships that could be construed as a potential conflict of interest.

## Footnotes

1 http://www.insect-genome.com/

2 https://web.expasy.org/protparam/

3 http://www.ncbi.nlm.nih.gov/Structure/cdd/wrpsb.cgi
